# The importance of the quadrivalent HPV vaccine in the elimination of cervical cancer in Brazil

**DOI:** 10.61622/rbgo/2024EDT04

**Published:** 2024-09-06

**Authors:** Cecília Martins Roteli-Martins, Ana Goretti Kalume Maranhão, Susana Cristina Aidé Viviani Fialho, Agnaldo Lopes da Silva-Filho

**Affiliations:** 1 Faculdade de Medicina do ABC Santo André SP Brazil Faculdade de Medicina do ABC, Santo André, SP, Brazil.; 2 Departamento do Programa Nacional de Imunizações Ministério da Saúde Brasília DF Brazil Departamento do Programa Nacional de Imunizações, Ministério da Saúde, Brasília, DF, Brazil.; 3 Universidade Federal Fluminense Niterói RJ Brazil Universidade Federal Fluminense, Niterói, RJ, Brazil.; 4 Universidade Federal de Minas Gerais Belo Horizonte MG Brazil Universidade Federal de Minas Gerais, Belo Horizonte, MG, Brazil.

The quadrivalent (Gardasil 4V, MSD) and bivalent (Cervarix 2V, GSK) human papillomavirus (HPV) vaccines were licensed globally between 2006 and 2008, and some Brazilian researchers contributed significantly to this achievement. Australia, the United Kingdom, Canada and Sweden soon began a comprehensive vaccination campaign, achieving broad and sustainable coverage in pre-adolescent and adolescent girls. Its results have been extensively published, demonstrating the impact on genital warts, high-grade cervical precursor lesions, and also on the incidence of cervical cancer.^1-3^

## HPV vaccination program in Brazil

The introduction of the quadrivalent HPV vaccine Gardasil 4V into the Brazilian National Immunization Schedule was approved by the National Committee for Technology Incorporation into the Unified Health System (Conitec) and incorporated into the National Immunization Schedule in 2014. This decision was based on a previous cost-effectiveness study that analyzed different scenarios for its introduction and on a favorable recommendation issued by the Technical Advisory Committee of the National Immunization Program (PNI). A partnership for technology transfer was established between the national laboratory Butantan and the laboratory Merck Sharp & Dohme to ensure the sustainability of the HPV vaccine.^4^

The vaccination strategy was initiated in March 2014 with the target population of girls aged 11-13 years and the priority vaccination location in public and private schools for the first dose (D1), with guidance for completing the vaccination schedule at Basic Health Units. The vaccination coverage target established was 90% with a vaccination schedule using three doses (first dose, second dose six months later and third dose 60 months after the first dose). In 2016, prior to the administration of the third dose in any age group, the schedule was changed to two doses in view of the publication of new studies showing no differences in antibody production between the two-dose and three-dose vaccination schedules.^5^

## Strategies to increase vaccination coverage

Since the introduction of the HPV vaccine into the Brazilian vaccination schedule, several strategies have been implemented to achieve the national target of 90% coverage. These include offering the vaccine in schools, having boys as a target group, gradually reducing the minimum age to nine years, and targeting specific populations, such as the immunocompromised, victims of violence, pre-exposure prophylaxis (PrEP) and post-exposure prophylaxis (PEP users), and more recently, those with recurrent respiratory papillomatosis, who receive a three-dose schedule.^6,7^

## Vaccine supply and the private sector

As result from the technology transfer and PDP (Partnership for Productive Development) process between the Butantan Institute and the Merck Sharp & Dohme laboratory, the Butantan Institute of São Paulo supplies the quadrivalent Gardasil vaccines to the Ministry of Health. The product is registered, imported and packaged by the Butantan Institute, and the Ministry of Health’s PNI distributes the doses to states and municipalities. Since 2019, the private sector has been selling Gardasil 9, which protects against the four HPV types present in Gardasil 4 (16, 18, 6 and 11) and five additional oncogenic HPV types (31, 33, 45, 52 and 58). Note that the private sector no longer sells the Gardasil 4 vaccine offered by the public sector. Since its implementation in the National Vaccination Schedule in 2014, more than 60 million doses of the Gardasil 4V have been distributed to all states of the Federation.^6,8^

## Global and national coverage challenges

In 2019, Brazil together with 193 countries committed to the World Health Organization (WHO) goal of achieving 90% HPV vaccination coverage in the adolescent population by 2030 in order to eliminate cervical cancer as a public health problem.^3^ Globally, the HPV vaccine coverage was higher among girls in high-income countries before and during the COVID-19 pandemic, compared to girls in low- and middle-income countries. During the pandemic, vaccination coverage among boys in high-income countries remained higher than that among girls in low- and middle-income countries. Globally, 23 countries have seen a sharp reduction in their HPV vaccination program (≥ 50% reduction in coverage), and in 2020-2021 compared to 2019, an additional 3.8 million girls did not receive even one dose of the HPV vaccine in countries with existing vaccination programs.^9^

In Brazil, even though safe and effective vaccines are provided free of charge by the Unified Health System (SUS), vaccination coverage remains far from the 90% target and varies between states. In 2023, average first-dose coverage rates were 75.8% for girls and 53.1% for boys, while second-dose coverage was 58.2% for girls and 33.1% for boys.^4^ Factors contributing to low coverage include the lack of public awareness of the importance and safety of the vaccine, including among healthcare workers, difficulties in access, and the spread of misinformation about vaccine safety, leading to fear and hesitancy about the vaccine among young people and their families.^10^

## Adoption of a single-dose vaccination strategy

Over the past decade, several studies have shown that a single dose of the HPV vaccine can provide similar protection to that of two- or three-dose schedules in certain age groups in areas with high vaccination coverage. This led the WHO in 2022 and the Pan American Health Organization (PAHO) in 2023 to adopt a single-dose HPV vaccination schedule. This decision was based on recommendations from its technical-scientific committees that suggested a single dose up to age 20, two doses at a six-month interval from age 21, and three doses for immunocompromised individuals. The countries were free to follow or not this recommendation.^6,11^

Therefore, considering the favorable results of increased coverage with HPV vaccination in countries that incorporated the single dose, in a recent decision, the PNI Department of the Ministry of Health adopted the recommendation of the Technical Advisory Committee of the PNI Department, HPV subgroup, of a single dose in accordance with recommendations of the PAHO and WHO. Note that the adoption of the single dose of HPV in the PNI will be for adolescents aged 9-14 years only, maintaining the recommendations for the other groups (immunosuppressed and victims of sexual violence).^6^

## Differences between public and private sector vaccines

There is ongoing debate about the differences between the public sector (4V) and private sector (9V) vaccines with respect to efficacy and safety. A recent systematic review included three randomized controlled trials: one in females aged 9–15 years (n = 600), one in females aged 16–26 years (n = 14,215), and one in males aged 16–26 years (n = 500). Results from the 16–26-year-old female cohort showed little or no difference in the incidence of high-grade cervical epithelial neoplasia, adenocarcinoma in situ, or cervical cancer between the Gardasil 4V and the Gardasil 9V HPV vaccines (odds ratio [OR]: 1.00, 95% confidence interval [CI]. The number of local adverse events was higher with the Gardasil 9V vaccine than with the Gardasil 4V vaccine (RR: 1.07, 95% CI), although without statistical significance.^12^

According to the MSD laboratory, 71 countries have already adopted the Gardasil 9V vaccine. However, the Brazilian Ministry of Health will continue to use the Gardasil 4V vaccine and will not recommend revaccination with the Gardasil 9V. The recent access to the Gardasil 9V, good news for the country, is considered for individual use in private clinics. However, for the public health program, there is no reason for this change, since the HPV types 16 and 18 contained in the Gardasil 4V vaccine represent more than 70% of all cases of cervical cancer worldwide, while the other types represent an additional of approximately 20%. The POP Brazil study, an epidemiological study on the prevalence of HPV infection in Brazil conducted by the Hospital Moinhos de Ventos and funded by the Ministry of Health revealed that HPV type 16 is the most prevalent high-risk subtype in our country. This study also showed that with the use of the Gardasil 4V vaccine, there was a greater than expected decrease in the prevalence of high-risk HPV types 16, 18, 45 and 52, the latter two present in the Gardasil 9V vaccine.^13^

Some international studies (United Kingdom and Sweden) have shown that cases of cervical cancer have reduced by more than 80% after vaccination with the Gardasil 4V.^14^

The current priority of the Ministry of Health is to increase coverage of the Gardasil 4V vaccine due to the difficulty in achieving the strategy of 90% coverage of girls up to 15 years of age. Among boys, the situation is more worrying, as the first dose is at 59.3% and the second dose at 37.1%. The Ministry of Health believes that the goal of achieving high coverage in the target audience (boys and girls aged 9-14 years) will have a much greater impact than expanding protection by type of vaccine.

## Strategies to overcome barriers

Strategies to overcome barriers are essential to ensure adherence and consequently increase coverage rates in programs. Public policies already begin with the adoption of single-dose vaccination schedules, offering the vaccine in schools and health centers, including flexible hours at vaccination centers, and reinforced recommendations from health professionals. By addressing these issues, we can work to improve HPV vaccination coverage, ensuring better protection of our young people against HPV-related diseases.

Differences in the formulations of HPV vaccines available in the public and private sectors can affect vaccine uptake and coverage rates. The public sector offers the Gardasil 4V, while the private sector provides Gardasil 9V, which covers additional oncogenic HPV types. However, this discrepancy should not influence public perception and access to comprehensive protection. Efforts to improve HPV vaccination coverage in Brazil include school-based programs, expanding the target population, and adopting a single-dose vaccination schedule as recommended by the WHO and PAHO. These initiatives aim to achieve the national target of 80% coverage and support the global goal of eliminating cervical cancer by 2030. Despite the availability of safe and effective vaccines, coverage in Brazil remains low and uneven across states. Public awareness, misinformation, and access challenges contribute to vaccine hesitancy. It is crucial to address these barriers through public health strategies and reinforce recommendations from healthcare providers. Adopting a single-dose vaccination schedule is a significant step toward increasing vaccine uptake. Continued efforts to promote awareness, ensure equitable access, and support healthcare providers are essential to achieve higher coverage rates and the reduction of HPV-related diseases. Understanding and addressing the factors that influence vaccine uptake will increase the effectiveness of HPV vaccination programs and protect future generations from HPV-related risks.

## References

[1] Garland SM, Cornall AM, Brotherton JM, Wark JD, Malloy MJ, Tabrizi SN (2018). Final analysis of a study assessing genital human papillomavirus genoprevalence in young Australian women, following eight years of a national vaccination program. Vaccine.

[2] Falcaro M, Castañon A, Ndlela B, Checchi M, Soldan K, Lopez-Bernal J (2021). The effects of the national HPV vaccination programme in England, UK, on cervical cancer and grade 3 cervical intraepithelial neoplasia incidence: a register-based observational study. Lancet.

[3] Silva AL, Roteli-Martins CM, Speck NM, Carvalho NS, Cândido EB, Teixeira JC (2024). The path to elimination: FEBRASGO 2023's targeted strategies against cervical cancer in Brazil. Rev Bras Ginecol Obstet.

[4] Ministério da Saúde (2014). SI-PNI - Sistema de Informação do Programa Nacional de Imunizações. Estratégia de Vacinação contra HPV. Coberturas vacinais - HPV Quadrivalente - Sexo feminino de 11 a 14 anos por idade e dose - Total Brasil - 2014.

[5] Ministério da Saúde, Secretaria Vigilância em Saúde, Departamento de Vigilância das Doenças Transmissíveis, Coordenação do Programa Nacional de Imunizações (2018). Informe técnico da ampliação da oferta das vacinas papilomavírus humano 6, 11, 16 e 18 (recombinante) - vacina HPV quadrivalente e meningocócica C (conjugada).

[6] Ministério da Saúde, Secretaria de Vigilância em Saúde e Ambiente, Departamento do Programa Nacional de Imunizações, Coordenação-Geral de Incorporação Científica e Imunização (2024). Nota Técnica No. 41/2024-CGICI/DPNI/SVSA/MS. Atualização das recomendações da vacinação contra HPV no Brasil.

[7] Oliveira IM, Martins BC, Soares LR (2024). Cobertura da vacina contra papilomavírus humano em Goiás. Epidemiol Serv Saúde.

[8] Gardasil 4: vacina papilomavírus humano 6, 11, 16 e 18 (recombinante).

[9] Casey RM, Akaba H, Hyde TB, Bloem P (2024). Covid-19 pandemic and equity of global human papillomavirus vaccination: descriptive study of World Health Organization-Unicef vaccination coverage estimates. BMJ Med.

[10] Nogueira-Rodrigues A, Flores MG, Macedo AO, Braga LA, Vieira CM, Sousa-Lima RM (2022). HPV vaccination in Latin America: coverage status, implementation challenges and strategies to overcome it. Front Oncol.

[11] World Health Organization (2021). Cervical cancer.

[12] Bergman H, Buckley BS, Villanueva G, Petkovic J, Garritty C, Lutje V (2019). Comparison of different human papillomavirus (HPV) vaccine types and dose schedules for prevention of HPV-related disease in females and males. Cochrane Database Syst Rev.

[13] Wendland EM, Kops NL, Bessel M, Comerlato J, Maranhão AG, Souza FM (2021). Effectiveness of a universal vaccination program with an HPV quadrivalent vaccine in young Brazilian women. Vaccine.

[14] Lei J, Ploner A, Elfström KM, Wang J, Roth A, Fang F (2020). HPV vaccination and the risk of invasive cervical cancer. N Engl J Med.

